# Enhancing mobile brain and body imaging: Open-source solutions for real-world research applications

**DOI:** 10.1016/j.isci.2026.116647

**Published:** 2026-07-07

**Authors:** Thorge Haupt, Paul Maanen, Mareike Daeglau, Miguel Contreras Altamirano, Anouk Sophie Stritzke, Franziska Kiene, Julius Welzel, Sarah Blum, Mandy Roheger, Stefan Debener

**Affiliations:** 1 Neuropsychology Lab, Department of Psychology, Carl von Ossietzky University of Oldenburg, Oldenburg, Germany; 2 Cluster of Excellence Hearing4, Carl von Ossietzky University of Oldenburg, Oldenburg, Germany; 3 Ambulatory Assessment in Psychology, Department of Psychology, Carl Von Ossietzky University Oldenburg, Oldenburg, Germany; 4 Neurogeriatrics, Department of Neurology, University Hospital Schleswig-Holstein, Kiel, Germany; 5 Cluster of Excellence Hearing4.connects, Carl von Ossietzky University of Oldenburg, Oldenburg, Germany

**Keywords:** mobile brain and body imaging, MoBi, lab streaming layer, LSL, multi-sensor, smartphone, GPS, google mediapipe

## Abstract

Mobile brain and body imaging (MoBI) requires monitoring human behavior in real-world settings using unobtrusive, portable hardware. To address multi-sensor signal streaming and time-synchronized recording on smartphones, we previously developed the open-source Android apps *Senda* and *Recorda*. *Senda* converts smartphone sensor signals into lab streaming layer (LSL) streams, while *Recorda* time-synchronizes and records them, bringing desktop LabRecorder functionality to android. Here, we report recent framework improvements. The ecosystem now integrates body-worn inertial measurement units (IMUs), GPS location streaming, and Google MediaPipe pose landmark motion capture. Furthermore, we improved usability by introducing real-time stream integrity feedback and Viewa, a new app for visualizing LSL streams. Two validation studies demonstrate reliable performance, showing high correlations between app-derived sensor data and reference sensors. These updates significantly expand research capabilities, seamlessly extending the LSL standard out of the traditional lab and into real-world scenarios.

## Highlights

• Open-source apps enable precise, time-synchronized mobile data streaming beyond the lab
• New features add onboard AI motion capture, audio classification, GPS, and external IMUs
• Live stream-integrity feedback and the new Viewa app improve usability
• The flexible framework turns off-the-shelf smartphones into research-grade recorder tools

## Graphical abstract

**Figure:**
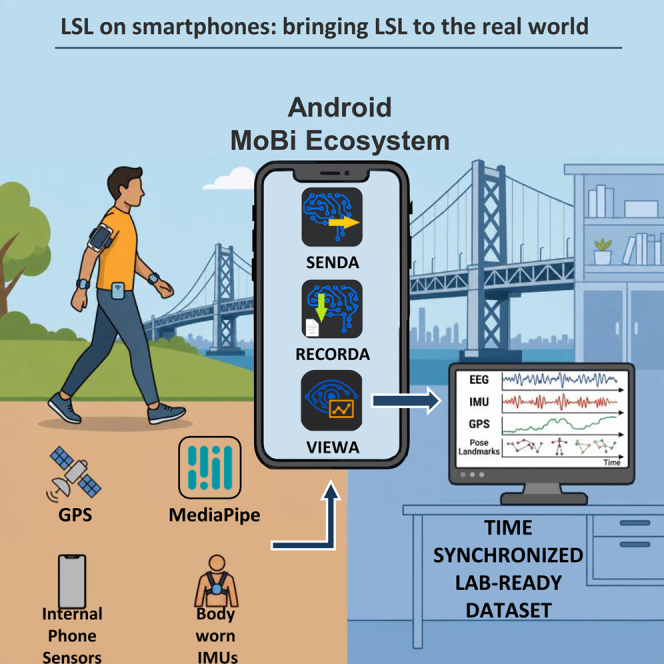

## Teaser

Software engineering; Open source software; Mobile computing

## Introduction

In recent years, there has been growing interest in investigating human behavior and cognition in real-world environments, beyond the confines of traditional laboratory settings.^1^^,^^2^^,^^3^ This shift aims to deepen our understanding of how contextual factors such as natural movement, social interaction, and complex multisensory environments shape cognitive and behavioral processes.^4^^,^^5^^,^^6^ For instance, examining the neural and cognitive demands associated with walking across varied natural terrains offers insights that extend beyond conventional treadmill-based cognitive-motor interaction.^7^ Reflecting this emphasis on context factors and ecological validity, the field has adopted the term mobile brain/body imaging (MoBI) to describe approaches that capture brain and body dynamics during naturalistic settings.^6^^,^^8^^,^^9^

While multimodal studies already present considerable challenges in controlled laboratory environments, implementing them in real-world contexts introduces even greater complexity.^10^^,^^11^ Two central technical challenges must be overcome to enable successful MoBI data acquisition. First, it is crucial to synchronously record signals from multiple sensing modalities (including neural, physiological, kinematic, location, video, and audio data) to fully capture the richness of behavior in dynamic, naturalistic environments. To solve this issue, the open-source project lab streaming layer (LSL; Kothe et al., 2024) has become a widely adopted standard in the MoBI community. Through community efforts, LSL provides both a way to stream data between devices and a rich ecosystem of at least 131 applications, ranging from Microsoft Xbox Controllers to electroencephalography (EEG) amplifiers from various companies (LSL-website, accessed 15.07.2025). To stream and synchronize data, the LSL framework implements a client-server architecture and assigns timestamps to each data sample using the device’s local clock. At the same time, it compensates for inter-device clock offsets using a network-based synchronization protocol, similar to the network time protocol. Together, this enables high-precision temporal alignment of multimodal data streams regardless of hardware platform or connection type, even in distributed setups. A detailed technical discussion of LSL’s synchronization mechanisms and performance is beyond the scope of this article and can be found in works such as Dasenbrock et al.,^12^ Iwama et al.,^13^ and LSL’s own documentation https://labstreaminglayer.readthedocs.io/.

The second technical challenge of multimodal, time-synchronized data collection lies in the devices for collecting and streaming data. Since multimodal measurement requires several different sensors being placed on or around the environment of the participant, ideally, recording should be possible with portable, lightweight, and unobtrusive hardware to avoid disrupting participants’ natural behavior. This demand benefits from advancements in wireless sensing and the miniaturization of mobile recording systems, paving the way for MoBI research. As a result, wireless EEG now enables neural data acquisition during activities such as outdoor walking,^7^^,^^14^ swimming,^15^ slacklining,^16^ naturalistic navigation,^17^ bungee jumping,^18^ and auditory attention in daily life settings.^1^^,^^19^^,^^20^^,^^21^^,^^22^ The same portable sensing infrastructure is accelerating mobile Health (mHealth) research, which uses mobile systems to support medical care, public health, and personal health management outside traditional clinical settings. These advancements benefit mobile interventions for Parkinson’s disease,^23^ hearing impairment,^24^^,^^25^ and cancer-related fatigue,^26^ as well as mobile diagnostic tools and assessment of symptom fluctuations over time and digital biomarkers for e.g., cognitive decline,^27^ depressive disorders,^28^ or post-COVID.^29^ Yet, assembling multi-sensor systems for such tasks remains non-trivial, since the interaction between different hardware setups needs to be tested beforehand, as well as conjoint analysis pipelines be validated. Here, time synchronization of different data streams, managed by LSL, is critical.

Balancing the breadth of natural behavior that can be recorded against the need for unobtrusive equipment remains a key challenge for MoBI. Advanced acquisition set-ups should therefore maximize both participant and device mobility.^30^ Smartphones offer an elegant solution to meet this demand. They are ubiquitous, lightweight, and familiar, yet already house high-quality inertial measurement units (IMUs), video, audio, and location sensors.^31^ Furthermore, recent on-device artificial intelligence (AI) solutions now support real-time video-based body-part tracking and audio classification,^32^^,^^33^^,^^34^ enabling applications ranging from tremor-frequency estimation for neurological assessment^35^ to mobile monitoring of navigation and movement patterns.^36^ Capitalizing on the potential of mobile phones as recording and streaming platform requires precise alignment of heterogeneous data streams. Adding to the LSL project,^37^ we developed two Android apps https://neuropsyol.github.io/: *Senda*, which broadcasts external and smartphone-sensor data as LSL streams, and *Recorda*, which captures time-synchronized multimodal recordings in the standard *.xdf* format.^38^ The two apps can be operated on a single device or on different devices that are connected to the same local area network, thereby integrating smartphones seamlessly into MoBI workflows.

**Figure 1:**
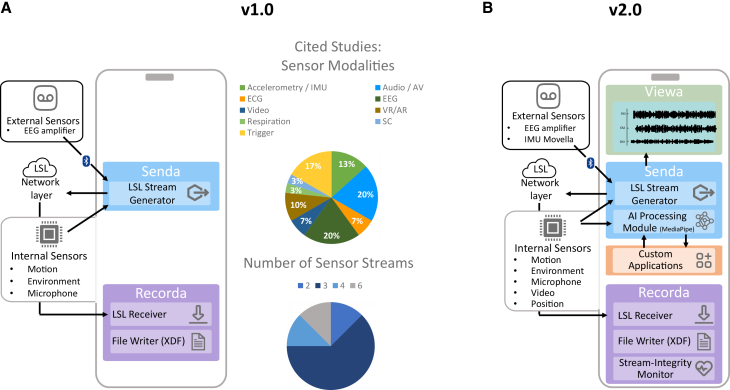
The general framework of *Senda* and *Recorda* v1.0 and v2.0, and the studies that have cited these applications (A) Shows the framework of *Senda* and *Recorda* V1.0 38. The pie chart in the middle shows the sensor modalities and the total number of sensors that were used in the studies that cited the original article. (B) Shows the updates that are presented in this study. Specifically, the integration of Movella sensors, position, and video settings as optional sensor streams. Furthermore, it highlights the Viewa functionality of visualizing the stream data. It also shows the integration of the AI processing module as well as the stream-integrity monitor.

Figure 1A illustrates the architecture of the original *Senda* and *Recorda* apps. To date, the apps have been used in at least eight peer-reviewed studies.^12^^,^^15^^,^^20^^,^^39^^,^^40^^,^^41^^,^^42^^,^^43^ As summarized in the pie charts in Figure 1A, the apps have supported the synchronized acquisition of a wide range of sensor modalities, underscoring their versatility. Among these studies, 13% incorporated accelerometry, 20% audio, 7% electrocardiography (ECG), 20% EEG, 20% trigger, 7% video, 10% virtual or augmented reality, 3% respiration, and 3% skin conductance.

Despite their initial utility, the first release of *Senda* and *Recorda* was limited in scope. The apps supported only a small set of onboard smartphone sensors, lacked built-in signal quality diagnostics, and did not accommodate studies requiring external motion-capture systems or real-time computer vision feedback. Here, we introduce two major upgrades to the open-source smartphone apps *Senda* and *Recorda* and briefly show how they can be implemented. First, we integrated Google MediaPipe https://github.com/google-ai-edge/mediapipe into the LSL workflow, enabling real-time, on-device AI-assisted functionality for pose estimation, gesture recognition, and audio-based inference. The code base can be freely accessed from the GitHub repo, and extensive documentation is available here: https://ai.google.dev/edge/mediapipe/solutions/guide. Second, we expanded the platform’s data integration capabilities by implementing (i) plug-and-play compatibility with state-of-the-art IMU sensor networks, (ii) continuous streaming of smartphone-based geolocation data, and (iii) a live stream-integrity monitoring feature. The latter allows the system to flag delayed or lost samples in real time, thus enabling researchers to monitor data quality continuously during data collection. We also present a new app named Viewa, which enables live monitoring of raw sensor signals. The new functionalities are illustrated in Figure 1B.

The remainder of this study is structured as follows. Section 2 describes codebase updates and improvements to the user interface (UI). Section 3 details the new sensor features for location tracking, motion sensing, and audio classification. In section 4, we demonstrate the utility of the upgraded platform through two ongoing studies: one investigating navigation in older adults with subjective cognitive decline (SCD), and another one capturing whole-body movement patterns and brain activity using a combination of body-worn IMUs, mobile EEG, and MediaPipe-based pose estimation. By offering a transparent, extensible, and open-source solution that runs on low-cost, standard hardware, we aim to facilitate the idea of pocketable labs, that is, investigations of human brain-behavior relationships in naturalistic settings.

### Design

#### Apps

This section summarizes software-level refinements to our core apps, *Recorda* and *Senda,* and introduces our new app, Viewa. Functional extensions, here the integration of MediaPipe, IMU, and GPS, are covered in Section 3.

#### Recorda v2.0

*Recorda* is our Android recorder that captures multiple LSL streams and writes them into a time-synchronized *.xdf* file. The overall architecture is unchanged, but we refactored the codebase for readability and improved UI usability. Further enhancements ensure compatibility with the latest smartphone models and new Android versions, which feature more stringent power management and security protocols. These adaptations include measures for persistent background mode and file system access for saving xdf files. We have opted to build these applications for the Android operating system due to the general ease of accessibility to the different sensors and large community efforts over iPhone Operating System.

**Figure 2:**
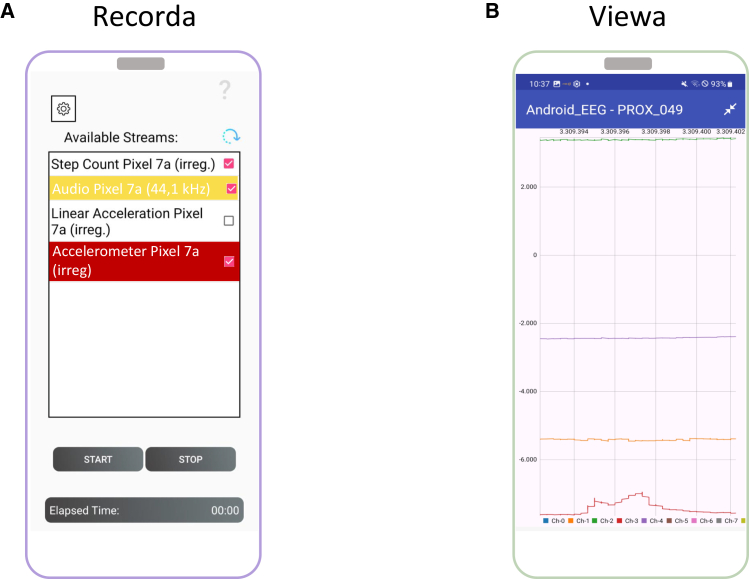
This figure shows the updates to the UI and the integration of the viewer functionality (A) Shows the different available streams. In case of lagging stream (i.e., no samples received for >1.5 s or sampling rate falling below 90% nominal sampling rate), the user is informed via notification, and the stream is colored yellow. When data loss (i.e., any exception while pulling data from LSL or no samples were received for > 7 s) occurs, the user also receives a notification, and the stream turns red. (B) Shows Viewa and a plot of the selected data streams.

Notable user interface updates include a new swipe-to-refresh feature for the stream list and a more intuitive and robust handling of permissions. To address potential data loss, which is a fundamental concern in wireless data recording scenarios, two significant modifications have been implemented. Firstly, before recording commences, *Recorda* now verifies the availability of selected streams and gives meaningful feedback in the UI. Secondly, during the recording, if a stream disconnects or the current sampling rate deviates from the nominal one, warnings and alerts indicating that the stream is less or non-responsive are shown (Figure 2). Specifically, *Recorda* compares the effective (measured) sampling rate against the nominal (declared) sampling rate using a fixed ratio threshold of 0.90, meaning the effective rate must not fall below 90% of the nominal rate before a “Laggy” warning is issued. This tolerance intentionally absorbs the small clock drifts typical of many sensors and also research-grade EEG. A complementary silence-based criterion is also applied: if no samples are received for 1.5 s, the stream is flagged as “Laggy” and after 7 s of continued silence, the status escalates to “not responding.” Streams declared with an irregular sampling rate (corresponding to LSL’s IRREGULAR_RATE) are exempt from rate-based assessment entirely, as no regular arrival of samples is expected for such streams. Currently, all quality thresholds are fixed constants in the source code and are not configurable at runtime. When setting up recording streams, users are advised to consider whether their recording streams require high or low precision. This may affect whether warnings of irregular streams can be considered as negligible or require detailed attention. Additionally, any exceptions encountered while pulling data from a stream result in immediate UI feedback by marking the respective stream and an error notification to the user (Figure 2). Both features are especially valuable in long-term mobile recordings, as they provide real-time warnings to prevent unnoticed data loss.

Brief dropouts on the sender side are absorbed by LSL’s internal buffer, and the affected samples are received in the subsequent call to pull data in a chunk. Since the sampling rate is measured over a 10-s look-back window, such transient gaps do not affect the quality indicator. However, sustained dropouts that exhaust the buffer on the sender site can cause samples to be silently discarded by LSL before they reach *Recorda*. In this case, the measured pull rate falls below 90% of the nominal rate, and a “Laggy” warning is issued. This quality indicator highlights a sustained throughout deficit rather than identifying specific missing samples, as *Recorda* does not perform individual sequence number verification. In short, the “Laggy” warning alerts the user that the recording is no longer maintaining real-time continuity, where the actual sampling rate deviates from the nominal one, resulting in potential data loss.

#### Senda v2.0

*Senda* publishes on-board smartphone sensors as LSL streams. The general architecture has been significantly refined to improve code quality, ease future maintenance, and enhance the overall robustness. In particular, the application has been adapted to comply with the stringent permission management and power-saving policies introduced in recent Android versions. Among other updates, measures have been taken to enable safe running in the background while accessing sensors continuously. Users can now operate other applications on the same smartphone, for example Viewa or experimental control applications while *Senda* and *Recorda* are running on the same device in the background.

Regarding sensor data handling, we have shifted from a fixed-rate polling model to an event-driven approach. *Senda* no longer queries sensors at a constant rate but instead reacts to callback functions initiated by the operating system when new sensor values are available. This aims to improve battery life and to better reflect actual sensor availability, reducing inaccuracies caused by mismatched sampling assumptions. Furthermore, this change acknowledges the inherent limitations of attempting to enforce regular sampling rates on systems where the operating system controls sensor access timings. The handling of the microphone is not affected by the transition to an event-driven approach, as audio capture requires a continuous isochronous stream at a fixed sampling rate of 44.1 kHz. Unlike motion sensors, where each event carries an explicit hardware-level timestamp allowing for offline correction of OS-induced jitter, audio samples rely on implicit timing based on their position in the sequence. Thus, any delivery delays or buffer overflows result in non-correctible gaps or phase shifts that cannot be reconstructed offline. Since Android 7, however, timestamp extraction for audio recordings is possible. Obtaining this information will enable offline correction of OS-induced jitter, but not the recovery of lost samples. In the current version of *Senda*, this has not been implemented so far, but will be fixed.

#### Viewa V1.0

Viewa is a new mobile application developed for ongoing visualization of incoming LSL streams (Figure 2). The App has no additional dependencies and operates on Android 11 or higher.

The primary functionality of Viewa is the discovery of available LSL data streams on the local network. Users can select streams and individual channels for visualization. The application supports dynamic autoscaling of plot axes to accommodate varying signal amplitudes and provides options for full-screen and multi-stream viewing. Visualization is implemented using the MPAndroidChart library https://github.com/PhilJay/MPAndroidChart. The UI is intended for easy inspection during experimental sessions and will soon be extended by the possibility of showing LSL markers alongside incoming time series data.

## Results

### New sensor features

This section details the integration of geolocation tracking, audio classification, and movement sensor data in the *Senda* framework. These updates are expected to significantly enhance the versatility of *Senda*, making it a more valuable tool in mobile sensor data acquisition and analysis.

#### Location tracking

Geolocation tracking plays a crucial role in mobile neuroscience research by linking brain activity to context in everyday settings.^44^^,^^45^ For example, there have been studies exploring the link between brain activity and architecture.^46^^,^^47^ In mHealth research, it enables the monitoring of physical activity and mobility patterns, which are essential for understanding the relationship between movement and health outcomes.^48^ For instance, geolocation tracking has been used to make estimations about the users’ mental health.^49^^,^^50^ Therefore, we have integrated location data from the smartphone as a new sensor stream, so that it can be analyzed in a MoBI setup, enhancing the utility of *Senda*.

**Figure 3:**
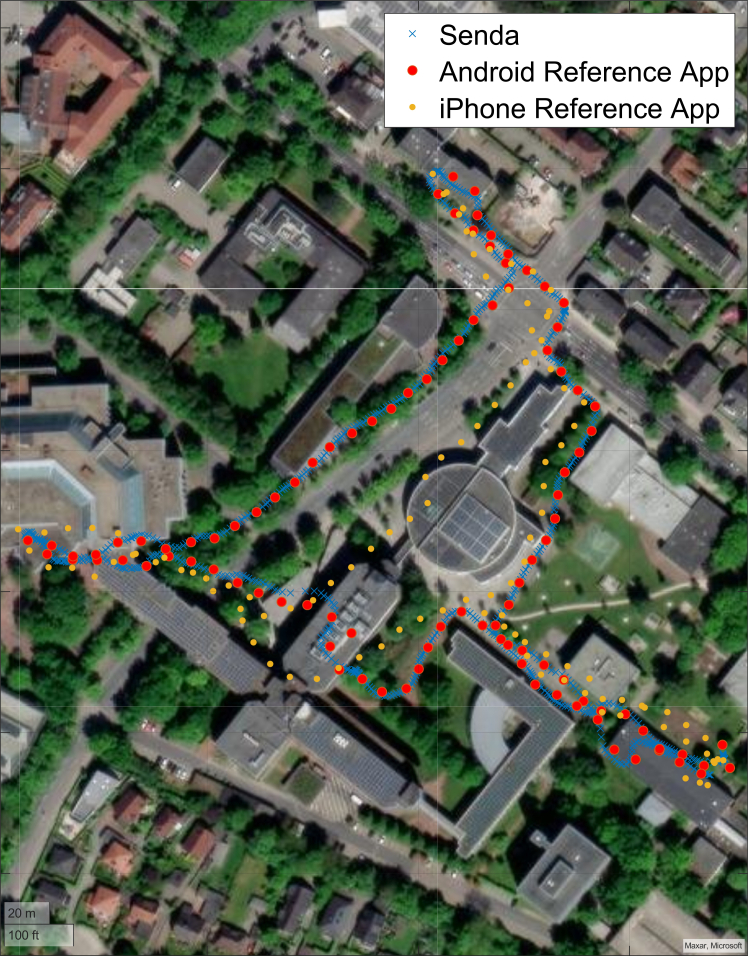
The track that was walked by a participant shows the geolocation points of the three GPS tracking sensors Blue is the *Senda* data, red is the android GPS tracker app, and yellow the track of the iPhone.

To showcase the implementation of *Senda*’s location stream, we compared the location data (using GPS, Galileo, BDS, and Glonass) emitted by *Senda* with the location data recorded by a commercial tracking app (Geo Tracker v5.3.3.3845) on the same smartphone and the location data recorded by a commercial app (GPS-Tracker Pro v1.8) on an iPhone (Figure 3).

The results showed that the mean absolute positional error was 1.4 m. Taking the position uncertainty given by the operating system into account yielded a mean deviation of *d*_*norm*_ = 0.31 (see Figure S1 for the error distribution).

The deviation lies well within normal GPS accuracy for smartphones in built-up areas. This measurement covered only one dataset obtained in one urban setting. Future work should profile *Senda*’s geolocation accuracy across diverse devices and environments (e.g., rural, indoor, and dense-high-rise areas). Our present integration of GPS tracking has shown that *Senda*’s geolocation stream seems sufficiently accurate to support a wide range of multimodal MoBI and mHealth studies.

It remains a matter of speculation why the reference track measured by the iPhone app deviates so greatly from the tracks measured by both Android apps, our app, and the Android commercial app. Possible reasons include additional filters not accessible by the UI or the inclusion of additional data not originating from the GPS signal. It is especially curious that the deviating track seems to “snap” to the boardwalk on the other side of the road, which might indicate an unprompted fusion with map data.

#### Audio classification

Investigating natural behavior, or cognition in response to various soundscapes, requires the concurrent capturing of sound events, raw audio, or general soundscape context. This is especially relevant for high-risk jobs, such as aviation, or to determine how noise impacts health and mental well-being.^51^^,^^52^ Specifically, the field of MoBI research stands to gain substantially from the implementation of audio classification techniques. The classification ensures privacy and reduces data redundancy, which is particularly relevant in public experiments where the inadvertent capture of bystander conversations in audio recordings is a concern.^41^ Here, privacy concerns necessitate the use of indirect measures of the acoustic environment.^53^

For the sound classification, the microphone’s audio stream captured with *Senda* is now also processed using the Google MediaPipe audio classifier https://ai.google.dev/edge/mediapipe/solutions/audio/audio_classifier. The classifier runs locally, never transmitting audio off the phone, and streams time-aligned label scores and change markers via LSL, so they can be analyzed alongside EEG, IMU, and GPS channels. This machine learning algorithm assesses audio input to assign scores across 521 different labels, enhancing data richness for applications requiring detailed audio analysis. Apart from the sound class categorization speech-to-text transcription is currently not possible.

After clock-alignment, the on- and off-line scores showed a mean label-score correlation of r ≈ 0.71 (highest, music = 0.8, lowest, silence = 0.6); the confusion matrix showed a mean accuracy of 81% (music 90%, speech 82%, vehicle 82%, silence 71%; Figure S2).

These results indicate that the on-device classifier preserves the accuracy of the offline implementation while operating in real time and without exporting privacy-sensitive audio. Future work will need to benchmark the classifier across a wider range of devices, noise conditions, and sound classes. Integrating MediaPipe audio classification in *Senda* is feasible and provides a privacy-considerate acoustic channel that can be combined with other sensor streams for multimodal recordings.

#### Motion sensing

While smartphones are increasingly popular for activity recognition in scientific research^54^ and can compete with body-worn IMUs for certain tasks,^55^ smartphones can currently not fully replace body-worn movement sensors. Body-worn sensors can be placed virtually anywhere on the body, allowing for strategic positioning on specific body parts to measure movements that a smartphone, typically carried in a pocket, cannot accurately capture.^56^ Therefore, integration of movement sensors into the data recording framework is crucial, as the position of body-worn sensors can be tailored to a specific biomechanical measurement.^57^ Integrating body-worn sensors into the same LSL stream lets researchers relate joint-level kinematics to simultaneously recorded sensor streams such as EEG, audio, or GPS data.

*Senda* now supports live Bluetooth streaming from Movella DOT body-worn IMUs, a versatile development platform for the capture of human kinematics (www.movella.com). Each DOT sends free-acceleration values, Euler angles, and time stamps that can be synchronized with a start pulse, ensuring millisecond alignment with the phone’s streams. Advanced sensor settings, such as sampling rate, filter profile, and custom name, can be configured in the Movella mobile app.

During a 327-s trial, the dual-DOT setup reliably streamed signals to *Senda* and *Recorda*. One IMU experienced a single burst of Bluetooth degradation (296 lost packets; 1.34%), which was identified in *Recorda* in real time, while the other sensor transmitted continuously. The intrinsic timing precision of the Movella sensors was high. The sensors’ inter-sample intervals deviated from the nominal 60 Hz sampling rate by only ≈12 μs rms and 7 μs rms, respectively (Figure S3), in other words, less than 0.1%. Next, we evaluated LSL and Movella timestamps concerning drift and jitter. The results showed that no drift occurred between the LSL and Movella timestamps and that jitter remained modest (11 and 13 ms rms, respectively; Figure S4).

We then compared the measurement values of the Movella sensors. The two DOT units produced virtually identical motion traces, as revealed by very high correlations of r ≥ 0.97 for acceleration magnitude and individual axes, and r ≥ 0.94 for yaw, pitch, and roll values.

When comparing the sensor values to the on-board IMU sensors, we found their acceleration magnitude aligned well with the phone’s own IMU (r = 0.91; angular-velocity correlation r = 0.88, limited by the phone gyro’s range).

These results suggest that *Senda* can integrate state-of-the-art body-worn IMUs into multimodal MoBI recordings. Packet loss was rare and correctable offline, but should be considered in latency-critical real-time applications. Full protocol and timing plots are provided in Supplement S3.

### Use cases

The previous sections detailed the *Senda* and *Recorda* updates and their contribution to the usage of the LSL protocol on mobile phones. In this section, we illustrate how the apps can be used in different experimental setups. In the first study illustrated below, it was required that the full body pose and specific body landmarks of individuals were tracked along with concurrent EEG and IMU recordings. The second study involves a navigation task where participants’ location, movement, and their interaction with a map on a smartphone were tracked concurrently.

#### Hand and pose landmark detection

AI-based pose and hand tracking add a visual-kinematic layer to our multimodal pipeline. Real-time landmark detection enables millisecond-accurate movement markers in natural settings, extending the scope of research beyond the constraints of a laboratory environment. This provides a foundation to uncover nuanced insights into the interplay of movement, cognition, and behavior. For instance, landmark detection data can be used to uncover how neurological disorders affect movement.^58^^,^^59^ Additionally, AI-based pose detection systems can provide researchers with detailed, quantitative analyses of movement patterns without the need for manual annotation.

**Figure 4:**
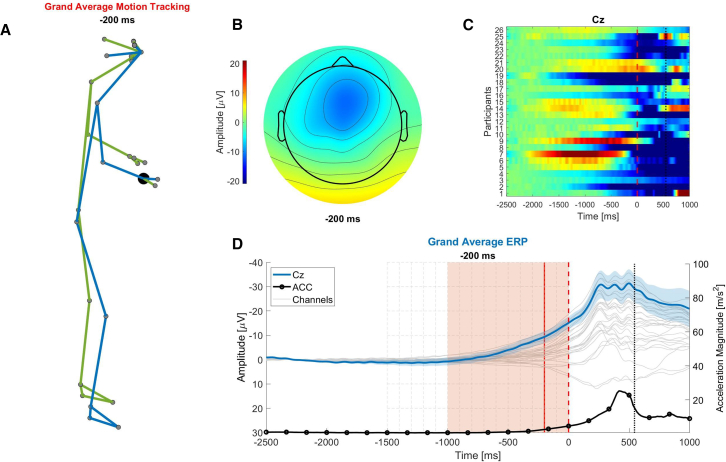
Visualizes the hand and pose landmarks as well as the RP during free throw shooting (A) Shows the output of the PLD. Here, the focus is the right wrist (black circle) and the eye landmarks. A marker is placed when the wrist surpasses eye level. (B) Shows the topography prior to wrist movement onset. (C) Displays the RP across participants at Cz channel. (D) The grand average RP (blue line) preceding voluntary actions. Movement onset (red dashed line) was determined from accelerometer signals (black line) attached to the wrist.

In our case, we needed to obtain specific, temporally precise data of landmarks during full-body movement. For this, we developed two custom applications: pose landmark and hand landmark detection, and used them alongside *Senda* and *Recorda*. The pose landmark application focuses on detecting the presence of a body within the camera stream and calculating the positions of significant body landmarks such as the nose, shoulders, and joints. To achieve this, we employed pose landmark detection (PLD) using Google MediaPipe https://ai.google.dev/edge/mediapipe/solutions/vision/pose_landmarker. The PLD system extracted real-time coordinates of 33 anatomical landmarks. The hand landmark application is designed to detect the presence and position of key landmarks of up to two hands within the camera stream (Figure 4A). It provides real-time data on hand orientation and finger positions, which are crucial for applications involving gesture recognition or fine motor skill analysis. These coordinates were streamed online via *Senda*.

These applications were utilized in a basketball free-throw study. The goal of this study was to determine whether brain activity could be predictive of hits or misses. In order to analyze the underlying neural marker, in this case the readiness potential (RP),^60^ which is associated with movement planning and initiation in EEG recordings, it was essential to establish precise event markers that aligned each trial relative to the onset of the movement (Figures 4B–4D). For movement onset detection, we specifically focused on the vertical (*y* axis) coordinates of the right wrist and right eye landmarks. An event marker was placed at the moment the wrist’s *y* coordinate surpassed that of the eye, corresponding to the upward motion initiating the free-throw. This operational definition of movement onset was applied uniformly across all trials and served as the temporal anchor point for aligning accelerometer signals and EEG data for subsequent analysis of preparatory neural activity.

#### Application of location tracking

Identifying individuals with an increased risk of developing Alzheimer’s disease is of paramount importance for the administration of interventions that are aimed at preventing or delaying the progression of dementia. SCD, characterized by a self-perceived decline in cognitive abilities without observable deficits in commonly used neuropsychological screening tests, constitutes such a risk factor.^61^ Given that these tests are not sensitive enough to identify SCD, the challenge lies in detecting possible subtle and early alterations in cognitive functions in older adults with SCD compared to those without.^62^

**Figure 5:**
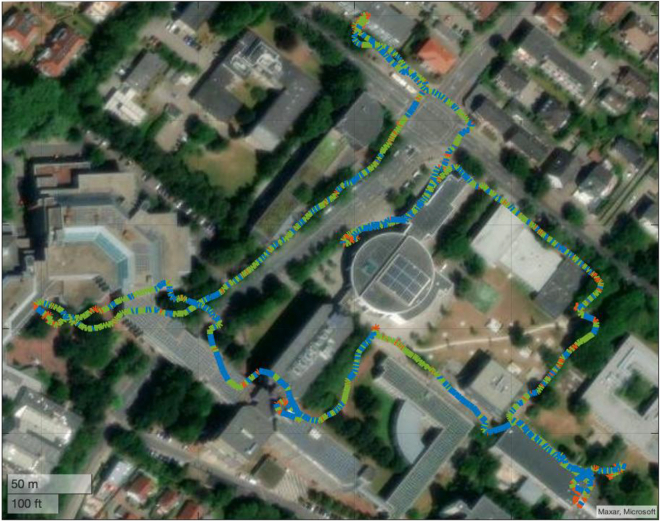
The track walked by one pilot subject during the navigation orientation study The colors represent different walking speeds, calculated based on the mean walking speed +–0.5∗standard deviation of this particular participant. Specifically, orange indicates slow (threshold, mean –0.5∗std), green mean (threshold, between fast and slow thresholds), and blue fast (threshold, mean +0.5∗std) walking segments.

Our study addresses this challenge by building on current findings that suggest individuals with SCD may experience difficulties in spatial orientation in comparison to age-matched individuals without SCD.^36^^,^^63^ To investigate this, we designed a wayfinding task set in an unfamiliar, naturalistic environment. Participants were required to locate and navigate to four target locations. Critically, our custom application tracks every interaction with the map, including opening and closing events, and sends out markers via LSL. These markers are synchronized with location and movement data obtained from wearable motion sensors (nine DOF IMUs from Movella), capturing comprehensive body motion signals along with corresponding GPS signals. This integration was not achievable with traditional map applications, which lack the capability to track user interactions and synchronize them with motion and location data. Consequently, our setup allows for unobtrusive capture of typical orientation behaviors, such as whole-body orientation stops and orientational head turns, thereby providing a more complete picture of the participants’ navigational behavior. Figure 5 shows the track walked by one pilot subject. The full analysis will include GPS data as well as map interactions and head and body movement data.

## Discussion

The collection of multimodal data is the key for understanding cognition and behavior in humans, not only in laboratory-based experiments but especially in everyday life. We have shown how the continuous development of the *Senda* and *Recorda* apps on smartphones enables time-synchronized multimodal recordings to achieve this goal. Specifically, the integration of the new features has significantly enhanced the functionality and reliability of the original apps. Importantly, through the integration of *Viewa,* data stream selection and integrity visualization are now feasible on the mobile phone, allowing continuous monitoring throughout the recording session. Furthermore, the open-source code allows for customization and integration of these tools seamlessly into existing MoBI setups, enabling adaptation to answer specific research questions. Our apps can be used together with and enrich existing setups by providing new possibilities for data streaming, recording, and visualization. This is feasible to complement stationary setups or realize completely mobile setups. This versatility and ease of integration underscore the critical role of open-source software in advancing scientific capabilities.

Based on reported usage of *Senda*/*Recorda,* we have upgraded the apps with onboard AI-based motion capture (MediaPipe), automated audio classification, continuous GPS location streaming, seamless ingestion of external IMU sensor streams, real-time stream-integrity feedback, and a refreshed UI. We have demonstrated the feasibility of integrating the novel features into the existing *Senda* and *Recorda* environments. Our analyses have shown the robustness and precision of the newly developed features.

The GPS tracking aligned well with commercial tracking apps and has been applied successfully in a navigational orientation task. Furthermore, our integration of the Google MediaPipe library for AI-based audio classification showed high agreement between the online and offline versions, underscoring how existing solutions can be implemented in our software. The integration of Movella DOT IMU sensors to *Senda* and *Recorda* marks a crucial step, as no other multimodal bluetooth-based IMU software is openly available. We have explored the use of this feature when collecting motion and GPS data. Overall, the new additions will expand the capabilities of researchers to capture more context variables, enabling naturalistic, real-time data collection beyond traditional laboratories.^31^ Furthermore, these features allow a wider range of mHealth applications to be developed.^64^^,^^65^

Besides the integration of new feature sensor streams, the open-source framework of *Senda* and *Recorda* allows for customization to fit experimental settings. The two studies we highlighted, one combining MediaPipe-based pose landmarking with mobile EEG recording, and the other investigating orientation behavior in adults with SCD, demonstrate the utility of our apps to be adopted to address specific research questions in naturalistic settings. Open-source software is indispensable for scientific research, particularly when customization and interoperability with existing lab setups are required. Proprietary solutions often fall short in providing the transparency and flexibility needed for rigorous scientific inquiry, due to license constraints or proprietary protocols and unavailable raw data. Open-source platforms, on the other hand, enable researchers to fully understand, modify, and extend software to meet specific experimental needs. Each feature that we have presented is available for immediate customization and integration into existing MoBI infrastructures, mobile and stationary. Together, they elevate an off-the-shelf smartphone to a research-grade, time-synchronized multimodal recorder, underscoring how open-source software can accelerate scientific capability, reproducibility, and foster a collaborative environment where innovations can be shared and improved upon by the community. This not only enhances the overall quality and reproducibility of data collection but also supports the principles of open science, promoting transparency and accessibility.

During the presentation of the new feature sensor streams, we provided brief examples illustrating their applications and how closely they align with reference measurements. As researchers adopt the *Senda* and *Recorda* framework, we encourage them to evaluate and report on three key aspects that are particularly relevant to MoBI research. First, long-term signal drift should be assessed, especially in recordings that span several hours. Second, while the LSL synchronizes clocks across devices, timing accuracy may still be affected by factors such as operating system scheduling, wireless communication delays (e.g., Wi-Fi, bluetooth), and the specific bridges used for external sensors. Therefore, every new system configuration should include a careful timing audit. Third, we urge researchers to report the hardware and software configurations used in their setups. As our testing was limited to a small number of Android handsets, documentation of successful applications across a broader range of devices and conditions will be invaluable to the MoBI community.

By addressing long-term drift, performing timing evaluations per setup, expanding compatibility across devices, and conducting modality-specific stress tests, the research community can significantly strengthen reproducibility and reliability. We invite researchers and developers to engage with our open-source software projects. Through shared use and collaborative development, we can collectively advance the field of MoBI, enhancing scientific transparency, accessibility, and global impact.^66^^,^^67^

With the updated version of *Senda* and *Recorda*, we have introduced several new features, extending the utility of LSL in the Android context. For instance, it is now possible to connect to Movella IMU sensors, as well as obtain video recordings as optional streams. Furthermore, we have improved monitoring of sensor streams with the extension of the stream-integrity monitor and the Viewa application. At last, the integration of the Google MediaPipe introduces an on-board AI processing module, increasing the versatility and direct extraction of features from sensor streams.

### Limitations of the study

For the implementation of GPS tracking our results indicate that *Senda* is able to reliably track and stream the user’s geolocation. However, it should be noted that a formal evaluation of *Senda* on all possible combinations of smartphones using a multitude of reference tracks was not conducted. Such an evaluation might not even be representative of the results, as end users might find that the results would be dependent on the local conditions, like the terrain buildup, the presence of Wi-Fi networks, or the density of cell phone towers.

Regarding the audio classification, it is unclear where the disagreements between online and offline classifiers come from as they both use the Yamnet audio classifier. One possible reason could be less than perfect temporal alignment between the audio file played back and the audio recording, which could lead to both classifier versions being fed slightly different audio snippets, which in turn leads to different scores, especially at the boundary between classes in the audio file.

At last, for the movement tracking, the Movella sensor data and the smartphone data were found to be in high agreement with each other. However, this statement cannot be generalized to all possible combinations of movement sensors and smartphones. A further limitation is that a comparison with the sensor data and a known ground truth, such as that provided by a movement plate, was not conducted. Another limitation is the experiment duration as we cannot exclude a slow sensor drift over hours of measurement. While our results are encouraging, they cannot replace the need for careful testing of the complete measurement setup by the end user.

## Resource availability

### Lead contact

Requests for further information and resources should be directed to and will be fulfilled by the lead contact, Thorge Haupt (thorge.lars.haupt@uni-oldenburg.de).

### Materials availability

This study did not generate new unique reagents.

### Data and code availability

• All data reported in this study will be shared by the lead contact upon request
• *Recorda*: https://github.com/NeuropsyOL/*RECORDA*, *Senda*: https://github.com/NeuropsyOL/*SENDA*, *Viewa*: https://github.com/NeuropsyOL/VIEWA
• Any additional information required to reanalyze the data reported in this study is available from the lead contact upon request.

Each repository includes detailed installation instructions and a list of software dependencies used during development and testing. While all three applications are designed to be ready-to-use out of the box, we strongly recommend performing timing validation within your specific setup to identify any potential deviations introduced by hardware or system configurations. The maintenance of the code base is currently secured for the next 7 years. For specific questions, we invite users to raise issues in GitHub.

As an open-source initiative, we also actively encourage researchers to contribute by validating newly supported sensor streams and sharing their own custom applications. These contributions will help advance *Senda* and *Recorda* as robust, community-driven platforms for MoBI research. For best practices and issues revolving around open source software we invite the reader to refer to Westner et al.^68^

## Acknowledgments

This work was funded by the 10.13039/501100001659Deutsche Forschungsgemeinschaft (10.13039/501100001659DFG, 10.13039/501100001659German Research Foundation) under Germany’s Excellence Strategy – EXC 2177/1 - project ID 390895286, and Germany’s Excellence Strategy – EXC 2177/2 - project ID 390895286.

## Author contributions

Conceptualization, S.D. and P.M.; methodology, P.M., T.H., and S.B.; software, P.M. and S.B.; validation, P.M. A.S.S., F.K., and M.C.A.; formal analysis, P.M. and T.H.; investigation, P.M., A.S.S., F.K., and M.C.A.; resources, S.D. and M.R.; data curation, P.M.; writing – original draft preparation, T.H. and P.M.; writing – review and editing, T.H., P.M., J.W., M.D., A.S.S., F.K., M.C.A., M.R., S.B., and S.D.; visualization, T.H. and P.M.; supervision, S.D. and M.R.; project administration, S.D.; funding acquisition, S.D.

## Declaration of interests

The authors declare no competing interests.

## Declaration of generative AI and AI-assisted technologies in the writing process

During the preparation of this work the authors used ChatGPT (v. 3o) and DeepL Write (academic style) to improve grammar and wording. After using these tools, the authors reviewed and edited the content as needed and take full responsibility for the content of the publication.

## STAR★Methods

### Key resources table

**Table:** 

REAGENT or RESOURCE	SOURCE	IDENTIFIER
**Software and algorithms**		

MATLAB (MathWorks) (2024a)	https://ch.mathworks.com/products/matlab.html	SCR_001622
Lab Steaming Layer	https://labstreaminglayer.org/#/	SCR_017631
Google MediaPipe	https://github.com/google-ai-edge/mediapipe	N/A
MP Android Chart	https://github.com/PhilJay/MPAndroidChart	N/A
Python 3.14.3	https://www.python.org/	SCR_008394
*RECORDA*	https://github.com/NeuropsyOL/*RECORDA*	https://doi.org/10.5281/zenodo.20589828
*SENDA*	https://github.com/NeuropsyOL/*SENDA*,	https://doi.org/10.5281/zenodo.20589828
VIEWA	https://github.com/NeuropsyOL/VIEWA	https://doi.org/10.5281/zenodo.20589828
Movella Xsens DOT	https://www.xsens.com/support/software-documentation	N/A
GEO-Tracker	https://geo-tracker.org/de/releases/	N/A
GPS Tracker Pro	https://apps.apple.com/de/app/gps-tracker-pro/id984920064	N/A

### Experimental model and study participant details

For the location tracking, and motion sensing were conducted by one of the authors. For the audio classification no human participant was needed. For the application of location tracking and the hand and pose landmark detection use cases, existing figures were presented respectively. For these, no original data was collected in the current study.

### Method details

#### Location tracking

For the geolocation we used three separate recording streams.

• **Test device (*Senda*):** Samsung S22+ (SM-S906B/DS), Android 14, *Senda* v2.0 (location callback at “HIGH_ACCURACY”), using GPS, Galileo, BDS, and Glonass
• **Reference #1:** Same handset running Geo Tracker v5.3.3.3845 (all filters disabled).
• **Reference #2:** iPhone 11 (MXD02ZD/A), iOS 17.4.1, GPS-Tracker Pro v1.8 (highest accuracy).

The comparison was performed for data collected during a about 15-min urban walk, with the two phones kept in the front pocket. The commercial apps were set to highest accuracy possible; all filters that were accessible via the UI were turned off. The data from the iPhone was removed, given the strong deviation from the other two GPS sensor streams. To access and analyze the data we followed these steps.

1. Exported KML tracks from Geo Tracker and GPS-Tracker Pro.
2. Upsampled commercial reference track by a factor of 100 to form quasi-continuous trajectories.
3. For each *Senda* sample, identified the closest point on each upsampled reference track and computed:

$$d_{abs}=\min\left|\left|p_{Senda}-p_{ref}\right|\right|$$

4. Normalised distance by Android-reported uncertainty:

$$d_{norm}=d_{abs}/\sigma_{GPS}$$

#### Audio classification

Freely available WAV files representing four classes were extracted: speech, vehicle, music, and silence. These samples were resampled to 16 kHz, converted to IEEE-float, cut into 5-s snippets and concatenated yielding a 130s track. For each class, that was not silence six, different snippets were used. The recording device Pixel 6, running *Senda* v 2.0, MediaPipe, and, *Recorda* v 2.0 for LSL logging. Playback was conducted on using a loudspeaker at normal volume inside an office room. Stimulus played once; *Senda* streamed the 521-element probability vectors and the on-board microphone recorded raw audio. A marker stream is generated whenever the leading label score changes, alongside a comprehensive 521-channel stream output approximately every second. The streams were stored as *.xdf*. Raw waveform was passed through the MediaPipe/YAMNet using a short python script. The output was stored as.txt file and loaded into MATLAB for comparsion to the online results. Since the exact start time of the online- and the offline classification are not exactly the same, due to a small, but unknown delay at the start of the online classification, we conducted temporal fine-alignment of the offline and online audio classifiers. For this, we applied the alignsignals function of MATLAB, which estimates the delay of one time series w.r.t. another time series by finding the delay that yields the highest correlation, on the score of the “speech” class. Classification outputs that did not match any of the four classes were set to other.

The recording was later analyzed offline with the same MediaPipe model, correlating the output scores as well as using a confusion matrix.

#### Motion sensing

For the motion sensing we used the following recording devices.

• **Phone:** Samsung S22 + (SM-S906B/DS), Android 14
• **IMUs:** Movella DOT “High-Performance” filter (36.30 × 30.35 × 10.80 mm and a weight of 11.2 g)
• **Mounting:** Sensors taped to rear shell of the phone; axes aligned with phone axes

To evaluate the precision of the IMU LSL stream, we strapped two Movella DOT IMUs to a Samsung S22 + and streamed their data to the phone via Bluetooth while performing a short protocol that combined normal walking with vigorous movements (e.g., jumping jacks and windmills). Our exemplary analysis focused on two timing metrics: drift, the slow clock divergence that can accumulate between sensors, and jitter, the random variation in the interval between successive samples. By examining both drift and jitter, we assessed whether the paired IMUs could provide time-consistent motion data suitable for integration with the phone’s streams.

### Quantification and statistical analysis

For the quantification and statistical analysis of the location tracking mean deviation was computed (Figure S1). For the audio classification Pearson correlations between actual and predicted classes, as well as a confusion matrix were obtained (Figure S2). For the motion sensing, mean and standard deviations were derived for the jitter (Figure S3) and drift (Figure S4) recordings.
