# Supplementary material for: Enhancing mobile brain and body imaging: Open-source solutions for real-world research applications
Source: iScience. 2026 Jul 7;29(7):116647. doi: 10.1016/j.isci.2026.116647 (PMC13356745; doi:10.1016/j.isci.2026.116647)
Supplement: Document S1. Figures S1–S4, and Table S1 [file mmc1.pdf]

iScience, Volume *29*

Supplemental information

Enhancing mobile brain and body imaging: Open-source solutions for real-world research applications

Thorge Haupt, Paul Maanen, Mareike Daeglau, Miguel Contreras Altamirano, Anouk Sophie Stritzke, Franziska Kiene, Julius Welzel, Sarah Blum, Mandy Roheger, and Stefan Debener

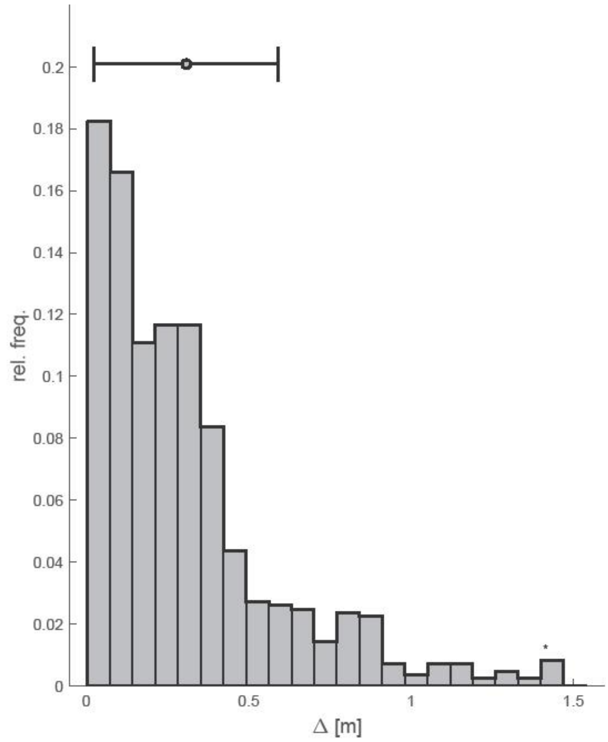

Figure S1. *Histogram showing the normalized deviation of the GPS tracks recorded by Senda and the Geo Tracker app. The mean deviation was* $d = 0.31$ *m. The star denotes the overflow bin.*

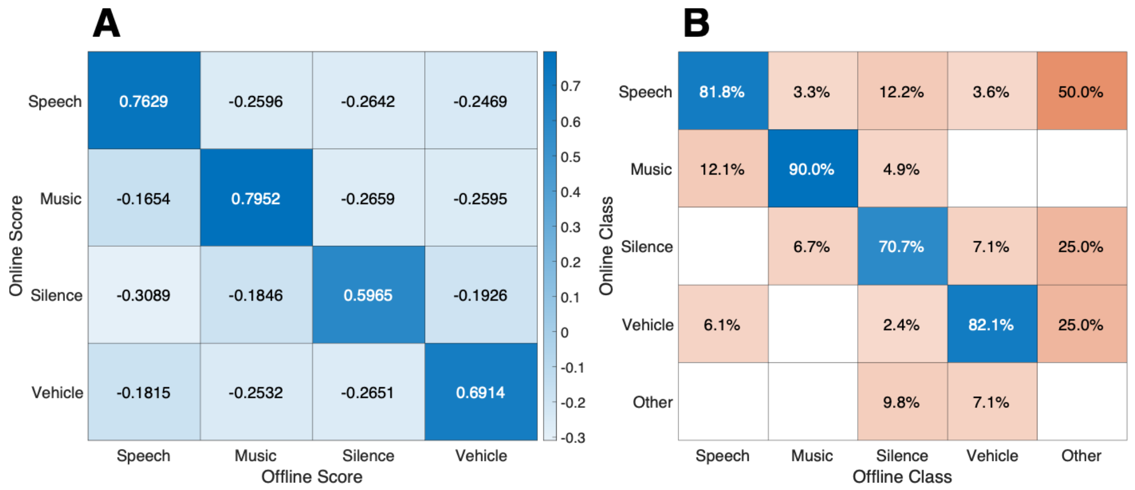

Figure S2. A: Pairwise correlation of the individual labels' scores. Only the sound classes included in the audio files are shown. Correlations on the main diagonal ranged between $r =$ 0.6 to $r = 0.8$. B: Confusion matrix comparing the most probable class emitted by offline and online classifiers for each point in time.

CLASS	R(SCORE)	TOP-LABEL AGREEMENT
**MUSIC**	0.80	90 %
**SPEECH**	0.76	82 %
**VEHICLE**	0.69	82 %
**SILENCE**	0.60	71 %

Table S1. contains the diagonal score correlations and top label agreements.

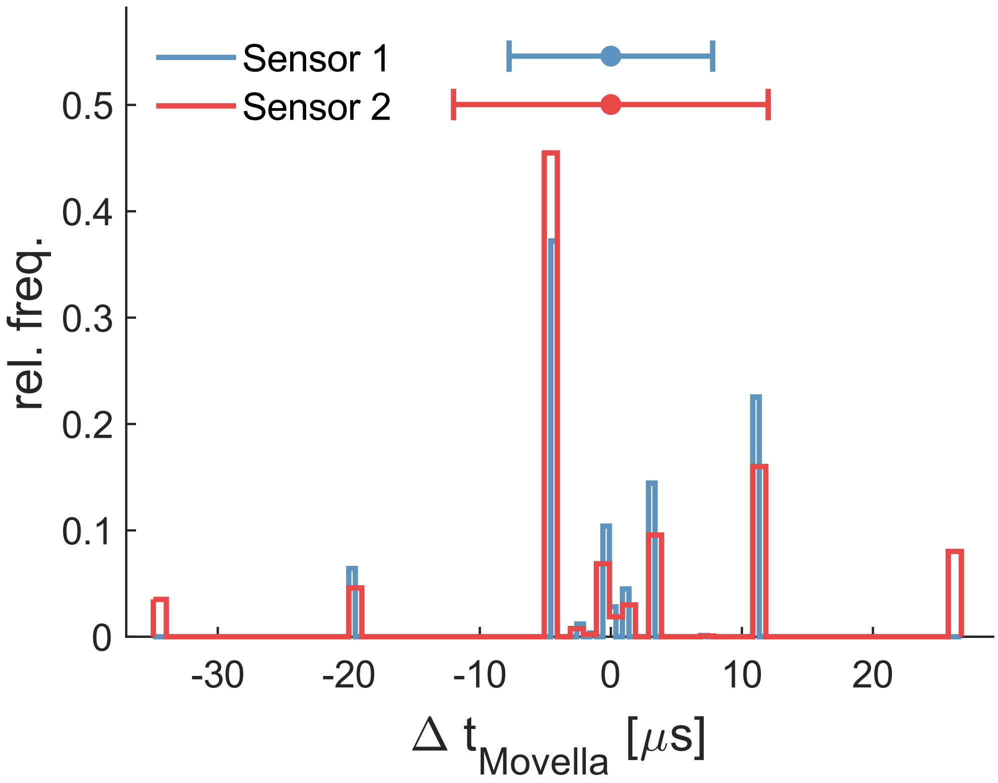

Figure S3: Shows the deviation of Sensor 1 (blue) and 2 (red) from the nominal sampling rate The bars on top show the mean (dot) and standard deviation of the jitter distribution, respectively.

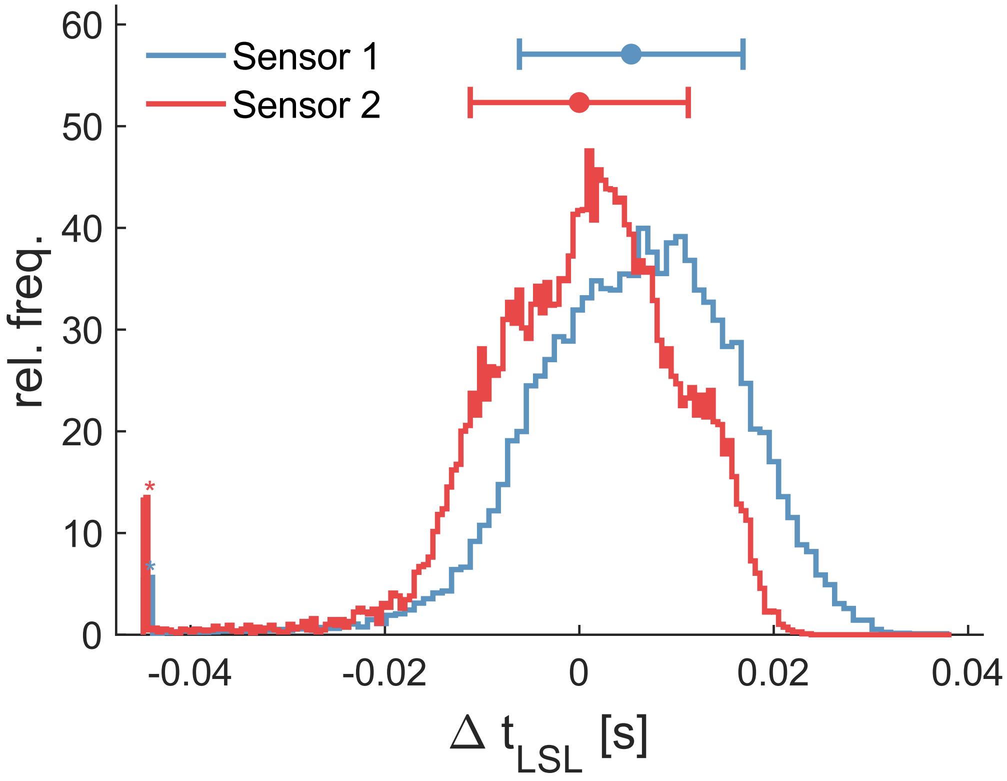

*Figure S4. Shows the distribution of jitter between the IMU sensor stream and the LSL stream for Sensor 1 (blue) and Sensor 2 (red), respectively. The bars on top denote the mean (dot) and standard deviation. The bins marked by * denote the over- and underflow bins. We measured an rms-width of the jitter distribution of* $(\Delta t_{LSL}) = 13$ *(B) The same for the other sensor. We found* $rms(\Delta t_{LSL}) = 11$
